# Bis{1-[(1*H*-benzimidazol-1-yl)meth­yl]-1*H*-imidazole-κ*N* ^3^}bis­(3,5-dicarb­oxy­benzoato-κ^2^ *O* ^1^,*O* ^1′^)nickel(II) octa­hydrate

**DOI:** 10.1107/S1600536813003322

**Published:** 2013-03-02

**Authors:** Yong-Yan Jia, Jing-Jing Fan, Xiang-Ge Yin, Wen-Long Zhao

**Affiliations:** a Pharmacy College, Henan University of Traditional Chinese Medicine, Zhengzhou 450008, People’s Republic of China

## Abstract

In the title complex, [Ni(C_9_H_5_O_6_)_2_(C_11_H_10_N_4_)_2_]·8H_2_O, the Ni^II^ ion exhibits site symmetry 2. It has a distorted octa­hedral coordination defined by two N atoms from two symmetry-related 1-[(1*H*-benzimidazol-1-yl)meth­yl]-1*H*-imidazole ligands and four O atoms from two symmetry-related 3,5-dicarb­oxy­benzoate anions. In the crystal, the complex mol­ecules and solvent water mol­ecules are linked *via* O—H⋯O, O—H⋯N and N—H⋯O hydrogen bonds, forming a three-dimensional structure. There are also a number of C—H⋯O inter­actions present.

## Related literature

For background information to Ni^II^ complexes constructed from both aromatic carboxyl­ates and *N*-heterocyclic ligands, see: Hu *et al.* (2011 ▶); Xu *et al.* (2010 ▶).
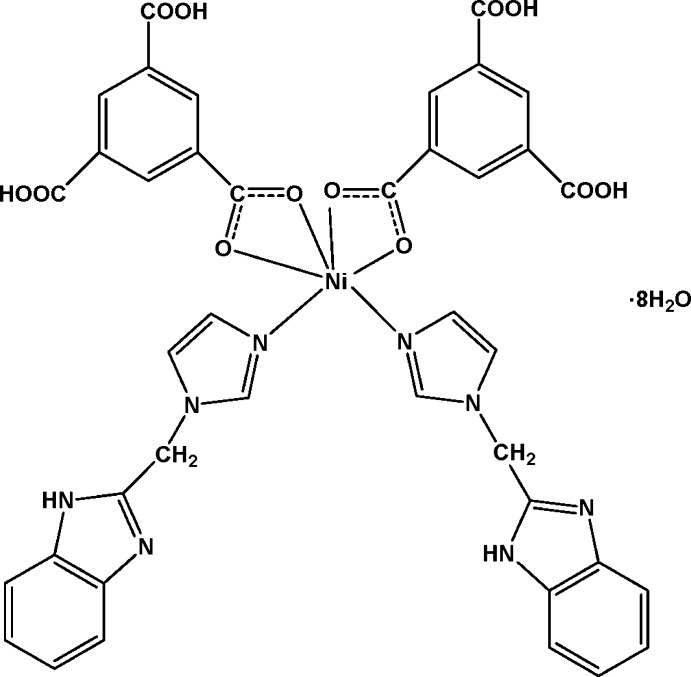


## Experimental

### 

#### Crystal data

[Ni(C_9_H_5_O_6_)_2_(C_11_H_10_N_4_)_2_]·8H_2_O
*M* *_r_* = 1017.56
Monoclinic, 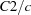

*a* = 20.623 (4) Å
*b* = 14.626 (3) Å
*c* = 15.471 (3) Å
β = 104.03 (3)°
*V* = 4527.2 (16) Å^3^
*Z* = 4
Mo *K*α radiation
μ = 0.52 mm^−1^
*T* = 293 K
0.19 × 0.17 × 0.12 mm

#### Data collection

Rigaku Saturn diffractometer
Absorption correction: multi-scan (*CrystalClear*; Rigaku/MSC, 2004 ▶) *T*_min_ = 0.908, *T*_max_ = 0.940
16185 measured reflections
4215 independent reflections
3719 reflections with *I* > 2σ(*I*)
*R*_int_ = 0.045

#### Refinement

*R*[*F* ^2^ > 2σ(*F* ^2^)] = 0.064
*wR*(*F* ^2^) = 0.172
*S* = 1.13
4215 reflections
314 parameters
H-atom parameters constrained
Δρ_max_ = 0.67 e Å^−3^
Δρ_min_ = −0.47 e Å^−3^

### 

Data collection: *CrystalClear* (Rigaku/MSC, 2004 ▶); cell refinement: *CrystalClear*; data reduction: *CrystalClear*; program(s) used to solve structure: *SHELXS97* (Sheldrick, 2008 ▶); program(s) used to refine structure: *SHELXL97* (Sheldrick, 2008 ▶); molecular graphics: *SHELXTL* (Sheldrick, 2008 ▶); software used to prepare material for publication: *publCIF* (Westrip, 2010 ▶).

## Supplementary Material

Crystal structure: contains datablock(s) global, I. DOI: 10.1107/S1600536813003322/su2557sup1.cif

Structure factors: contains datablock(s) I. DOI: 10.1107/S1600536813003322/su2557Isup2.hkl

Additional supplementary materials:  crystallographic information; 3D view; checkCIF report

## Figures and Tables

**Table 1:** Hydrogen-bond geometry (Å, °)

*D*—H⋯*A*	*D*—H	H⋯*A*	*D*⋯*A*	*D*—H⋯*A*
O7—H1*W*⋯O10	0.85	2.09	2.936 (7)	170
O7—H2*W*⋯O6^ii^	0.85	1.96	2.802 (4)	172
O3—H3⋯O7^iii^	0.82	2.04	2.782 (5)	150
N3—H3*B*⋯O8^iv^	0.86	1.95	2.780 (5)	162
O8—H3*W*⋯O1^v^	0.85	2.05	2.866 (4)	161
O8—H4*W*⋯O7	0.85	2.14	2.915 (5)	151
O5—H5⋯N4^vi^	0.82	1.77	2.586 (4)	172
O9—H5*W*⋯O3^vii^	0.85	2.28	3.133 (5)	180
O9—H6*W*⋯O6^i^	0.85	2.05	2.791 (5)	146
O10—H7*W*⋯O4	0.85	1.73	2.579 (7)	173
C1—H1*A*⋯O5^vii^	0.93	2.56	3.320 (4)	139
C3—H3*A*⋯O4^v^	0.93	2.46	3.078 (5)	124
C4—H4*A*⋯O1^viii^	0.97	2.55	3.479 (4)	160

Symmetry codes: (i) 
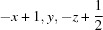
; (ii) 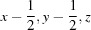; (iii) 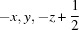; (iv) 
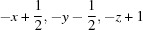
; (v) 
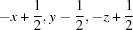
; (vi) 
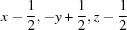
; (vii) 
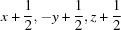
; (viii) 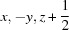.

**Fig. 1.**
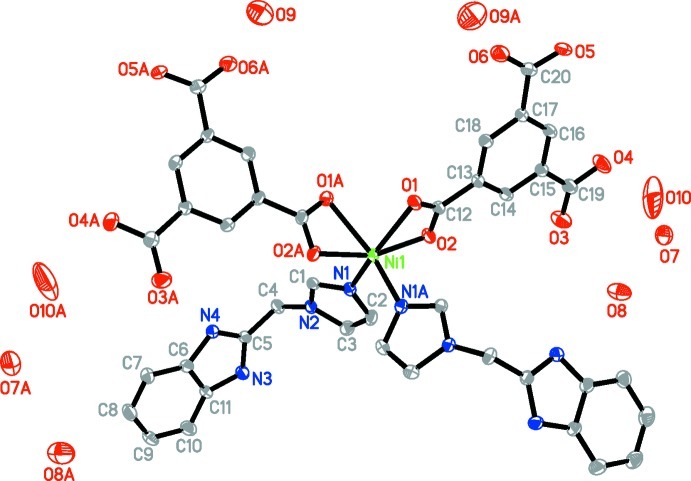
View of the molecular structure of the title complex, showing the atom labelling. Displacement ellipsoids are drawn at the 30% probability level. H atoms have been omitted for clarity [symmetry code: A = -x + 1, y, -z + 1/2.].

## supplementary crystallographic information

### Comment

Since Ni^II^ ions are able to coordinate simultaneously to both oxygen and nitrogen containing ligands the final products can exhibit attractive structures and useful functional properties. A great number of Ni^II^ complexes containing both aromatic carboxylates and N-heterocyclic ligands have been reported (Hu *et al.*, 2011; Xu *et al.*, 2010). In order to further explore such compounds with possibly new structures, we selected 1-((1*H*-benzimidazol-1-yl)methyl)-1*H*-imidazole and 1,3,5-benzenetricarboxylic acid as ligands to self-assemble with Ni(NO_3_)_2_ and obtained the title complex. The crystal structure of which is reported on herein.

As shown in Fig. 1, the Ni^II^ ion is located on a two-fold rotation axis. Each Ni^II^ ion features a distorted octahedral geometry and is hexacoordinated by four O atoms from two symmetry related 1,3,5-dicarboxybenzoate anions, in which carboxylate groups coordinate to the Ni^II^ ion in the chelating mode, and by two N atoms from two symmetry related 1-((1*H*-benzimidazol-1-yl)methyl)- 1*H*-imidazole ligands, which coordinate to Ni^II^ ion in a monodentate mode. Atoms O2, O2A, O1A, N1A, and Ni1 are nearly co-planar (the mean deviation from the plane is 0.0726 (12) Å), while atom O1 and N1 are located in the apical positions.

In the crystal, a series of O—H···O, O—H···N, and N—H···O hydrogen bonds linking, solvent water to water molecules, solvent water molecules and carboxylate O atoms, carboxyl groups and benzimidazole N atoms, and benzimidazole units and solvent water molecules, consolidate the crystal packing forming a three-dimensional structure (Table 1). There are also a number of C-H···O interactions present (Table 1).

### Experimental

A mixture of Ni(NO_3_)_2_ (0.1 mmol), 1-((1*H*-benzimidazol-1-yl)methyl)-1*H*-imidazole (0.1 mmol), 1,3,5-benzenetricarboxylic acid (0.1 mmol) and water (10 ml) was placed in a 25 ml Teflon-lined stainless steel vessel and heated at 373 K for 72 h, then cooled to room temperature. Green crystals were obtained from the filtrate and dried in air.

### Refinement

The C-bound H atoms were positioned geometrically and refined as riding atom: C-H = 0.93 (aromatic) Å and 0.97 (CH_2_) Å. The water and NH H atoms were located in difference Fourier maps. In the final cycles of refinement they were included in calculated positions and refined as riding atoms: N-H = 0.86 Å and O-H = 0.82 (OH) Å and O-H = 0.85 (H_2_O) Å. All H atoms were refined with U_iso_(H) = 1.2U_eq_(C,N,O).

### Crystal data

**Table:**

[Ni(C_9_H_5_O_6_)_2_(C_11_H_10_N_4_)_2_]·8H_2_O	*F*(000) = 2120
*M**_r_* = 1017.56	*D*_x_ = 1.493 Mg m^−^^3^
Monoclinic, *C*2/*c*	Mo *K*α radiation, λ = 0.71073 Å
*a* = 20.623 (4) Å	Cell parameters from 5823 reflections
*b* = 14.626 (3) Å	θ = 1.7–27.9°
*c* = 15.471 (3) Å	µ = 0.52 mm^−^^1^
β = 104.03 (3)°	*T* = 293 K
*V* = 4527.2 (16) Å^3^	Prism, green
*Z* = 4	0.19 × 0.17 × 0.12 mm

### Data collection

**Table:**

Rigaku Saturn diffractometer	4215 independent reflections
Radiation source: fine-focus sealed tube	3719 reflections with *I* > 2σ(*I*)
Graphite monochromator	*R*_int_ = 0.045
Detector resolution: 28.5714 pixels mm^-1^	θ_max_ = 25.5°, θ_min_ = 2.0°
ω scans	*h* = −24→24
Absorption correction: multi-scan (*CrystalClear*; Rigaku/MSC, 2004)	*k* = −14→17
*T*_min_ = 0.908, *T*_max_ = 0.940	*l* = −18→15
16185 measured reflections	

### Refinement

**Table:**

Refinement on *F*^2^	Primary atom site location: structure-invariant direct methods
Least-squares matrix: full	Secondary atom site location: difference Fourier map
*R*[*F*^2^ > 2σ(*F*^2^)] = 0.064	Hydrogen site location: inferred from neighbouring sites
*wR*(*F*^2^) = 0.172	H-atom parameters constrained
*S* = 1.13	*w* = 1/[σ^2^(*F*_o_^2^) + (0.0894*P*)^2^ + 4.146*P*] where *P* = (*F*_o_^2^ + 2*F*_c_^2^)/3
4215 reflections	(Δ/σ)_max_ < 0.001
314 parameters	Δρ_max_ = 0.67 e Å^−^^3^
0 restraints	Δρ_min_ = −0.47 e Å^−^^3^

### Special details

**Table:**

Geometry. Bond distances, angles etc. have been calculated using the rounded fractional coordinates. All su's are estimated from the variances of the (full) variance-covariance matrix. The cell esds are taken into account in the estimation of distances, angles and torsion angles
Refinement. Refinement of *F*^2^ against ALL reflections. The weighted *R*-factor *wR* and goodness of fit *S* are based on *F*^2^, conventional *R*-factors *R* are based on *F*, with *F* set to zero for negative *F*^2^. The threshold expression of *F*^2^ > σ(*F*^2^) is used only for calculating *R*-factors(gt) *etc*. and is not relevant to the choice of reflections for refinement. *R*-factors based on *F*^2^ are statistically about twice as large as those based on *F*, and *R*- factors based on ALL data will be even larger.

### Fractional atomic coordinates and isotropic or equivalent isotropic displacement parameters (Å^2^)

**Table:**

	*x*	*y*	*z*	*U*_iso_*/*U*_eq_	
Ni1	0.50000	0.05398 (4)	0.25000	0.0263 (2)	
O1	0.44973 (11)	0.17115 (16)	0.15907 (16)	0.0365 (8)	
O2	0.40111 (11)	0.07735 (16)	0.23391 (16)	0.0338 (8)	
O3	0.15958 (15)	0.0697 (2)	0.1857 (2)	0.0608 (13)	
O4	0.09844 (14)	0.1905 (2)	0.1294 (3)	0.0686 (13)	
O5	0.20784 (14)	0.46292 (18)	0.0447 (2)	0.0522 (10)	
O6	0.31803 (15)	0.46684 (19)	0.0667 (2)	0.0568 (10)	
N1	0.51386 (13)	−0.03683 (19)	0.35162 (18)	0.0300 (9)	
N2	0.54931 (13)	−0.09621 (19)	0.48450 (18)	0.0293 (8)	
N3	0.65040 (14)	−0.2528 (2)	0.61442 (19)	0.0340 (9)	
N4	0.70616 (14)	−0.13469 (19)	0.58371 (19)	0.0327 (9)	
C1	0.55815 (16)	−0.0300 (2)	0.4289 (2)	0.0277 (10)	
C2	0.47484 (17)	−0.1117 (2)	0.3587 (2)	0.0377 (11)	
C3	0.49601 (18)	−0.1488 (3)	0.4409 (2)	0.0390 (12)	
C4	0.58605 (17)	−0.1066 (2)	0.5776 (2)	0.0335 (11)	
C5	0.64692 (16)	−0.1648 (2)	0.5901 (2)	0.0292 (10)	
C6	0.75139 (17)	−0.2070 (2)	0.6066 (2)	0.0326 (10)	
C7	0.81932 (18)	−0.2116 (3)	0.6135 (2)	0.0413 (11)	
C8	0.8498 (2)	−0.2936 (3)	0.6411 (3)	0.0488 (14)	
C9	0.8142 (2)	−0.3693 (3)	0.6597 (3)	0.0491 (14)	
C10	0.7470 (2)	−0.3652 (3)	0.6525 (3)	0.0473 (14)	
C11	0.71606 (17)	−0.2822 (2)	0.6260 (2)	0.0329 (11)	
C12	0.39819 (16)	0.1458 (2)	0.1831 (2)	0.0305 (10)	
C13	0.33326 (16)	0.1947 (2)	0.1540 (2)	0.0298 (10)	
C14	0.27601 (17)	0.1527 (2)	0.1660 (2)	0.0338 (11)	
C15	0.21442 (16)	0.1966 (2)	0.1419 (2)	0.0330 (11)	
C16	0.21099 (17)	0.2849 (2)	0.1080 (2)	0.0352 (11)	
C17	0.26850 (17)	0.3292 (2)	0.0971 (2)	0.0331 (11)	
C18	0.32924 (17)	0.2827 (2)	0.1189 (2)	0.0325 (10)	
C19	0.15416 (19)	0.1473 (3)	0.1539 (3)	0.0441 (14)	
C20	0.26497 (19)	0.4268 (2)	0.0666 (3)	0.0403 (11)	
O7	−0.07964 (17)	−0.0247 (2)	0.2232 (2)	0.0719 (11)	
O8	−0.03597 (17)	−0.1763 (2)	0.3458 (3)	0.0795 (14)	
O9	0.5992 (2)	0.5579 (3)	0.5235 (3)	0.107 (2)	
O10	−0.0070 (3)	0.0946 (5)	0.1264 (4)	0.183 (3)	
H1A	0.59100	0.01480	0.44280	0.0330*	
H2A	0.43950	−0.13330	0.31400	0.0450*	
H3	0.12890	0.06020	0.20980	0.0730*	
H3A	0.47820	−0.19940	0.46330	0.0470*	
H3B	0.61750	−0.28560	0.62170	0.0410*	
H4A	0.55640	−0.13310	0.61090	0.0400*	
H4B	0.59910	−0.04650	0.60230	0.0400*	
H5	0.21090	0.51760	0.05690	0.0630*	
H7A	0.84320	−0.16180	0.60010	0.0500*	
H8A	0.89570	−0.29890	0.64760	0.0580*	
H9A	0.83670	−0.42380	0.67730	0.0590*	
H10A	0.72310	−0.41550	0.66490	0.0570*	
H14A	0.27870	0.09430	0.19040	0.0410*	
H16A	0.17000	0.31480	0.09230	0.0420*	
H18A	0.36750	0.31080	0.11000	0.0390*	
H1W	−0.06060	0.01530	0.19800	0.0860*	
H2W	−0.11320	−0.02630	0.17870	0.0860*	
H3W	−0.00440	−0.21200	0.34160	0.0950*	
H4W	−0.04180	−0.14410	0.29870	0.0950*	
H5W	0.61540	0.52330	0.56760	0.1280*	
H6W	0.63240	0.55220	0.50050	0.1280*	
H7W	0.02720	0.12840	0.13100	0.2200*	
H8W	−0.03660	0.09890	0.07760	0.2200*	

### Atomic displacement parameters (Å^2^)

**Table:**

	*U*^11^	*U*^22^	*U*^33^	*U*^12^	*U*^13^	*U*^23^
Ni1	0.0205 (3)	0.0263 (4)	0.0307 (4)	0.0000	0.0033 (2)	0.0000
O1	0.0243 (12)	0.0331 (14)	0.0513 (15)	0.0031 (10)	0.0077 (11)	0.0034 (11)
O2	0.0270 (12)	0.0347 (14)	0.0377 (13)	0.0067 (10)	0.0042 (10)	0.0067 (11)
O3	0.0446 (18)	0.0437 (18)	0.102 (3)	0.0041 (13)	0.0330 (17)	0.0223 (16)
O4	0.0281 (15)	0.0512 (19)	0.124 (3)	0.0069 (13)	0.0135 (16)	0.0257 (18)
O5	0.0395 (16)	0.0236 (13)	0.087 (2)	0.0071 (11)	0.0026 (14)	0.0013 (14)
O6	0.0411 (17)	0.0366 (16)	0.091 (2)	−0.0024 (13)	0.0129 (15)	0.0155 (15)
N1	0.0242 (14)	0.0300 (15)	0.0347 (16)	0.0026 (11)	0.0048 (12)	0.0012 (12)
N2	0.0243 (14)	0.0289 (15)	0.0332 (15)	0.0024 (11)	0.0039 (11)	0.0034 (12)
N3	0.0287 (15)	0.0293 (16)	0.0445 (17)	−0.0005 (12)	0.0101 (13)	0.0083 (13)
N4	0.0280 (15)	0.0285 (15)	0.0395 (16)	0.0000 (12)	0.0044 (12)	0.0006 (12)
C1	0.0224 (15)	0.0256 (17)	0.0349 (18)	0.0017 (13)	0.0067 (13)	0.0007 (13)
C2	0.0286 (18)	0.037 (2)	0.043 (2)	−0.0081 (15)	0.0002 (15)	0.0003 (16)
C3	0.036 (2)	0.032 (2)	0.047 (2)	−0.0066 (15)	0.0064 (16)	0.0048 (16)
C4	0.0305 (18)	0.037 (2)	0.0316 (18)	0.0079 (15)	0.0049 (14)	0.0020 (15)
C5	0.0297 (17)	0.0279 (18)	0.0280 (17)	0.0025 (13)	0.0029 (13)	0.0005 (13)
C6	0.0335 (18)	0.0285 (18)	0.0341 (18)	0.0037 (14)	0.0048 (14)	−0.0005 (14)
C7	0.0310 (19)	0.045 (2)	0.047 (2)	0.0018 (16)	0.0079 (16)	−0.0029 (17)
C8	0.034 (2)	0.056 (3)	0.055 (2)	0.0137 (18)	0.0083 (17)	−0.003 (2)
C9	0.044 (2)	0.040 (2)	0.060 (3)	0.0183 (18)	0.0065 (19)	0.0026 (19)
C10	0.045 (2)	0.035 (2)	0.061 (3)	0.0074 (17)	0.0112 (19)	0.0092 (19)
C11	0.0298 (18)	0.0283 (18)	0.0387 (19)	0.0059 (14)	0.0044 (14)	0.0016 (14)
C12	0.0264 (17)	0.0296 (18)	0.0324 (18)	0.0049 (14)	0.0012 (13)	−0.0053 (14)
C13	0.0272 (17)	0.0275 (18)	0.0325 (18)	0.0050 (13)	0.0030 (13)	−0.0008 (14)
C14	0.0286 (17)	0.0263 (18)	0.045 (2)	0.0036 (14)	0.0061 (15)	0.0034 (15)
C15	0.0262 (17)	0.0280 (18)	0.044 (2)	0.0009 (14)	0.0067 (15)	0.0008 (15)
C16	0.0262 (17)	0.0310 (19)	0.047 (2)	0.0073 (14)	0.0062 (15)	0.0017 (15)
C17	0.0269 (17)	0.0274 (18)	0.042 (2)	0.0052 (13)	0.0023 (14)	−0.0004 (14)
C18	0.0278 (17)	0.0298 (18)	0.0400 (19)	0.0018 (13)	0.0082 (14)	−0.0026 (15)
C19	0.034 (2)	0.036 (2)	0.061 (3)	0.0022 (16)	0.0092 (17)	0.0035 (18)
C20	0.038 (2)	0.0285 (19)	0.053 (2)	0.0036 (15)	0.0081 (17)	0.0001 (16)
O7	0.0484 (19)	0.075 (2)	0.090 (2)	−0.0035 (17)	0.0122 (17)	−0.0016 (19)
O8	0.074 (2)	0.059 (2)	0.125 (3)	0.0071 (18)	0.062 (2)	0.008 (2)
O9	0.114 (4)	0.104 (4)	0.106 (3)	0.019 (3)	0.034 (3)	0.002 (3)
O10	0.087 (4)	0.270 (8)	0.165 (5)	−0.095 (5)	−0.024 (4)	0.106 (5)

### Geometric parameters (Å, º)

**Table:**

Ni1—O1	2.299 (2)	N3—H3B	0.8600
Ni1—O2	2.023 (2)	C2—C3	1.354 (4)
Ni1—N1	2.025 (3)	C4—C5	1.490 (5)
Ni1—O1^i^	2.299 (2)	C6—C11	1.392 (4)
Ni1—O2^i^	2.023 (2)	C6—C7	1.381 (5)
Ni1—N1^i^	2.025 (3)	C7—C8	1.373 (6)
O1—C12	1.264 (4)	C8—C9	1.397 (6)
O2—C12	1.265 (4)	C9—C10	1.365 (6)
O3—C19	1.231 (5)	C10—C11	1.386 (5)
O4—C19	1.285 (5)	C12—C13	1.488 (5)
O5—C20	1.260 (5)	C13—C18	1.392 (4)
O6—C20	1.241 (5)	C13—C14	1.383 (5)
O3—H3	0.8200	C14—C15	1.391 (5)
O5—H5	0.8200	C15—C16	1.389 (4)
O7—H1W	0.8500	C15—C19	1.487 (5)
O7—H2W	0.8500	C16—C17	1.397 (5)
O8—H3W	0.8500	C17—C18	1.393 (5)
O8—H4W	0.8500	C17—C20	1.500 (4)
O9—H5W	0.8500	C1—H1A	0.9300
O9—H6W	0.8500	C2—H2A	0.9300
O10—H8W	0.8500	C3—H3A	0.9300
O10—H7W	0.8500	C4—H4A	0.9700
N1—C1	1.321 (4)	C4—H4B	0.9700
N1—C2	1.379 (4)	C7—H7A	0.9300
N2—C4	1.464 (4)	C8—H8A	0.9300
N2—C3	1.377 (5)	C9—H9A	0.9300
N2—C1	1.337 (4)	C10—H10A	0.9300
N3—C5	1.338 (4)	C14—H14A	0.9300
N3—C11	1.390 (5)	C16—H16A	0.9300
N4—C6	1.398 (4)	C18—H18A	0.9300
N4—C5	1.325 (4)		
			
O1—Ni1—O2	60.46 (9)	C6—C11—C10	121.9 (3)
O1—Ni1—N1	157.24 (10)	N3—C11—C10	132.3 (3)
O1—Ni1—O1^i^	83.59 (9)	O1—C12—O2	119.9 (3)
O1—Ni1—O2^i^	103.95 (10)	O1—C12—C13	122.0 (3)
O1—Ni1—N1^i^	93.21 (10)	O2—C12—C13	118.2 (3)
O2—Ni1—N1	98.90 (11)	C12—C13—C14	118.7 (3)
O1^i^—Ni1—O2	103.95 (10)	C14—C13—C18	119.4 (3)
O2—Ni1—O2^i^	160.54 (10)	C12—C13—C18	121.8 (3)
O2—Ni1—N1^i^	93.84 (11)	C13—C14—C15	120.9 (3)
O1^i^—Ni1—N1	93.21 (10)	C14—C15—C19	118.6 (3)
O2^i^—Ni1—N1	93.84 (11)	C16—C15—C19	122.0 (3)
N1—Ni1—N1^i^	98.01 (12)	C14—C15—C16	119.3 (3)
O1^i^—Ni1—O2^i^	60.46 (9)	C15—C16—C17	120.6 (3)
O1^i^—Ni1—N1^i^	157.24 (10)	C16—C17—C20	120.1 (3)
O2^i^—Ni1—N1^i^	98.90 (11)	C16—C17—C18	119.0 (3)
Ni1—O1—C12	83.57 (18)	C18—C17—C20	120.9 (3)
Ni1—O2—C12	96.0 (2)	C13—C18—C17	120.7 (3)
C19—O3—H3	109.00	O3—C19—O4	124.0 (4)
C20—O5—H5	109.00	O3—C19—C15	120.0 (4)
H1W—O7—H2W	91.00	O4—C19—C15	116.1 (4)
H3W—O8—H4W	103.00	O6—C20—C17	118.3 (3)
H5W—O9—H6W	94.00	O5—C20—O6	124.7 (3)
H7W—O10—H8W	116.00	O5—C20—C17	117.0 (3)
Ni1—N1—C2	127.4 (2)	N2—C1—H1A	125.00
Ni1—N1—C1	126.1 (2)	N1—C1—H1A	125.00
C1—N1—C2	106.1 (3)	N1—C2—H2A	125.00
C1—N2—C4	126.1 (3)	C3—C2—H2A	125.00
C1—N2—C3	107.9 (3)	N2—C3—H3A	127.00
C3—N2—C4	125.8 (3)	C2—C3—H3A	127.00
C5—N3—C11	108.5 (3)	N2—C4—H4B	109.00
C5—N4—C6	107.5 (3)	C5—C4—H4A	109.00
C11—N3—H3B	126.00	C5—C4—H4B	109.00
C5—N3—H3B	126.00	N2—C4—H4A	109.00
N1—C1—N2	110.8 (3)	H4A—C4—H4B	108.00
N1—C2—C3	109.4 (3)	C8—C7—H7A	122.00
N2—C3—C2	105.9 (3)	C6—C7—H7A	122.00
N2—C4—C5	113.9 (3)	C9—C8—H8A	119.00
N3—C5—N4	110.7 (3)	C7—C8—H8A	119.00
N3—C5—C4	124.9 (3)	C10—C9—H9A	119.00
N4—C5—C4	124.3 (3)	C8—C9—H9A	119.00
N4—C6—C7	131.2 (3)	C9—C10—H10A	122.00
C7—C6—C11	121.3 (3)	C11—C10—H10A	122.00
N4—C6—C11	107.5 (3)	C13—C14—H14A	120.00
C6—C7—C8	116.5 (4)	C15—C14—H14A	120.00
C7—C8—C9	122.2 (4)	C17—C16—H16A	120.00
C8—C9—C10	121.5 (4)	C15—C16—H16A	120.00
C9—C10—C11	116.6 (4)	C13—C18—H18A	120.00
N3—C11—C6	105.8 (3)	C17—C18—H18A	120.00
			
O2—Ni1—O1—C12	1.55 (17)	C5—N4—C6—C11	0.7 (3)
N1—Ni1—O1—C12	−25.5 (3)	N1—C2—C3—N2	−0.6 (4)
O1^i^—Ni1—O1—C12	−108.47 (18)	N2—C4—C5—N3	−99.5 (4)
O2^i^—Ni1—O1—C12	−165.85 (18)	N2—C4—C5—N4	84.7 (4)
N1^i^—Ni1—O1—C12	94.15 (19)	N4—C6—C7—C8	177.7 (4)
O1—Ni1—O2—C12	−1.55 (17)	C11—C6—C7—C8	−0.6 (5)
N1—Ni1—O2—C12	168.19 (19)	N4—C6—C11—N3	−0.2 (3)
O1^i^—Ni1—O2—C12	72.61 (19)	N4—C6—C11—C10	−179.1 (3)
N1^i^—Ni1—O2—C12	−93.1 (2)	C7—C6—C11—N3	178.5 (3)
O1—Ni1—N1—C1	−101.9 (3)	C7—C6—C11—C10	−0.4 (5)
O1—Ni1—N1—C2	70.1 (4)	C6—C7—C8—C9	1.2 (6)
O2—Ni1—N1—C1	−125.5 (3)	C7—C8—C9—C10	−0.9 (7)
O2—Ni1—N1—C2	46.5 (3)	C8—C9—C10—C11	−0.1 (6)
O1^i^—Ni1—N1—C1	−20.8 (3)	C9—C10—C11—N3	−177.8 (4)
O1^i^—Ni1—N1—C2	151.2 (3)	C9—C10—C11—C6	0.8 (6)
O2^i^—Ni1—N1—C1	39.8 (3)	O1—C12—C13—C14	166.0 (3)
O2^i^—Ni1—N1—C2	−148.3 (3)	O1—C12—C13—C18	−16.9 (5)
N1^i^—Ni1—N1—C1	139.4 (3)	O2—C12—C13—C14	−14.5 (4)
N1^i^—Ni1—N1—C2	−48.7 (3)	O2—C12—C13—C18	162.6 (3)
Ni1—O1—C12—O2	−2.5 (3)	C12—C13—C14—C15	178.4 (3)
Ni1—O1—C12—C13	177.0 (3)	C18—C13—C14—C15	1.2 (5)
Ni1—O2—C12—O1	2.8 (3)	C12—C13—C18—C17	−176.1 (3)
Ni1—O2—C12—C13	−176.7 (2)	C14—C13—C18—C17	1.0 (5)
Ni1—N1—C1—N2	173.3 (2)	C13—C14—C15—C16	−2.1 (5)
C2—N1—C1—N2	−0.1 (4)	C13—C14—C15—C19	178.3 (3)
Ni1—N1—C2—C3	−172.8 (2)	C14—C15—C16—C17	0.7 (5)
C1—N1—C2—C3	0.4 (4)	C19—C15—C16—C17	−179.7 (3)
C3—N2—C1—N1	−0.3 (4)	C14—C15—C19—O3	0.9 (6)
C4—N2—C1—N1	−175.6 (3)	C14—C15—C19—O4	−179.1 (4)
C1—N2—C3—C2	0.5 (4)	C16—C15—C19—O3	−178.7 (4)
C4—N2—C3—C2	175.9 (3)	C16—C15—C19—O4	1.3 (6)
C1—N2—C4—C5	−91.3 (4)	C15—C16—C17—C18	1.4 (4)
C3—N2—C4—C5	94.2 (4)	C15—C16—C17—C20	−175.7 (3)
C11—N3—C5—N4	0.9 (4)	C16—C17—C18—C13	−2.3 (4)
C11—N3—C5—C4	−175.4 (3)	C20—C17—C18—C13	174.8 (3)
C5—N3—C11—C6	−0.4 (3)	C16—C17—C20—O5	−5.8 (5)
C5—N3—C11—C10	178.3 (4)	C16—C17—C20—O6	172.5 (3)
C6—N4—C5—N3	−1.0 (4)	C18—C17—C20—O5	177.2 (3)
C6—N4—C5—C4	175.3 (3)	C18—C17—C20—O6	−4.5 (5)
C5—N4—C6—C7	−177.7 (3)		

Symmetry code: (i) −*x*+1, *y*, −*z*+1/2.

### Hydrogen-bond geometry (Å, º)

**Table:**

*D*—H···*A*	*D*—H	H···*A*	*D*···*A*	*D*—H···*A*
O7—H1*W*···O10	0.85	2.09	2.936 (7)	170
O7—H2*W*···O6^ii^	0.85	1.96	2.802 (4)	172
O3—H3···O7^iii^	0.82	2.04	2.782 (5)	150
N3—H3*B*···O8^iv^	0.86	1.95	2.780 (5)	162
O8—H3*W*···O1^v^	0.85	2.05	2.866 (4)	161
O8—H4*W*···O7	0.85	2.14	2.915 (5)	151
O5—H5···N4^vi^	0.82	1.77	2.586 (4)	172
O9—H5*W*···O3^vii^	0.85	2.28	3.133 (5)	180
O9—H6*W*···O6^i^	0.85	2.05	2.791 (5)	146
O10—H7*W*···O4	0.85	1.73	2.579 (7)	173
C1—H1*A*···O5^vii^	0.93	2.56	3.320 (4)	139
C3—H3*A*···O4^v^	0.93	2.46	3.078 (5)	124
C4—H4*A*···O2^viii^	0.97	2.49	2.895 (4)	105
C4—H4*A*···O1^ix^	0.97	2.55	3.479 (4)	160

Symmetry codes: (i) −*x*+1, *y*, −*z*+1/2; (ii) *x*−1/2, *y*−1/2, *z*; (iii) −*x*, *y*, −*z*+1/2; (iv) −*x*+1/2, −*y*−1/2, −*z*+1; (v) −*x*+1/2, *y*−1/2, −*z*+1/2; (vi) *x*−1/2, −*y*+1/2, *z*−1/2; (vii) *x*+1/2, −*y*+1/2, *z*+1/2; (viii) −*x*+1, −*y*, −*z*+1; (ix) *x*, −*y*, *z*+1/2.
